# Exploring Body Composition Profiles in Patients with Gastrointestinal Cancers: A Cross-Sectional Analysis of the NUTRISCREEN Project

**DOI:** 10.3390/curroncol33100591

**Published:** 2026-09-30

**Authors:** Assunta Luongo, Francesco Pio Maria Di Carlo, Sergio Coluccia, Paola Rocco, Natalia Russo, Emanuela Racca, Maria D’Amico, Elisabetta Coppola, Davide D’Errico, Anna Crispo, Valeria Turrà, Piera Maiolino, Francesca Foschini, Lucrezia Silvestro, Anna Nappi, Carmela Romano, Antonio Avallone, Sandro Pignata, Giuseppe Porciello

**Affiliations:** 1 Epidemiology and Biostatistics Unit, Istituto Nazionale Tumori IRCCS “Fondazione G. Pascale”, 80131 Naples, Italy; assunta.luongo@istitutotumori.na.it (A.L.); francescopio.dicarlo@istitutotumori.na.it (F.P.M.D.C.); paola.rocco@istitutotumori.na.it (P.R.); natalia.russo@istitutotumori.na.it (N.R.); a.crispo@istitutotumori.na.it (A.C.); 2 Experimental Clinical Abdominal Oncology Unit, Istituto Nazionale Tumori IRCCS “Fondazione G. Pascale”, 80131 Naples, Italy; emanuela.racca@istitutotumori.na.it (E.R.); francesca.foschini@istitutotumori.na.it (F.F.); l.silvestro@istitutotumori.na.it (L.S.); a.nappi@istitutotumori.na.it (A.N.); c.romano@istitutotumori.na.it (C.R.); a.avallone@istitutotumori.na.it (A.A.); 3 Colorectal Surgical Oncology Abdominal Oncology Department, Istituto Nazionale Tumori IRCCS “Fondazione G. Pascale”, 80131 Naples, Italy; m.damico@istitutotumori.na.it; 4 Department of Urology and Gynecology, Istituto Nazionale Tumori IRCCS “Fondazione G. Pascale”, 80131 Naples, Italy; elisabetta.coppola@istitutotumori.na.it (E.C.); davide.derrico@istitutotumori.na.it (D.D.); s.pignata@istitutotumori.na.it (S.P.); 5 Dietetics and Artificial Nutrition, Istituto Nazionale Tumori IRCCS “Fondazione G. Pascale”, 80131 Naples, Italy; v.turra@istitutotumori.na.it; 6 Pharmacy Unit, Istituto Nazionale Tumori IRCCS “Fondazione G. Pascale”, 80131 Naples, Italy; p.maiolino@istitutotumori.na.it

**Keywords:** gastrointestinal cancers, body composition, malnutrition risk, nutritional status assessment, health-related quality of life

## Abstract

Body composition (BC) analysis may integrate assessment of nutritional risk in patients with gastrointestinal cancers. Applying unsupervised machine learning techniques, we derived BC profiles (BCPs) and investigated their association with malnutrition risk and quality of life. This cross-sectional analysis includes gastrointestinal cancer patients enrolled in the NUTRItional and sarcopenia SCREENing project, part of the ONCOCAMP study (ClinicalTrials.gov ID: NCT06270602). Malnutrition assessment was performed using the NRS-2002, anthropometry and BC analysis; HRQoL was evaluated with the EORTC QLQ-C30 questionnaire. Principal component analysis and k-means clustering identified BCPs. Data from 189 patients (mean age: 66.3 ± 11.2 years) with gastric (37%), pancreatic (26.5%), rectal (24.3%) and hepatocellular carcinoma (12.2%) were analyzed. Overall, 36.5% of patients were at nutritional risk (NRS-2002 ≥ 3). Two BC clusters, High Muscle Profile (HMP) and Low Muscle Profile (LMP), were derived. Although no statistically significant association was observed in the overall cohort, multivariable analysis stratified by gender identified an association between LMP and nutritional risk among male patients (OR = 3.77, 95% CI: 1.08–14.3, *p* = 0.042); however, this finding should be considered exploratory. Nutritional risk, but not BC profiles, was associated with poorer Summary Score (β = 8.0, 95% CI: −14 to −2.3, *p* = 0.007) and higher Symptom Score (β = 9.8, 95% CI:4.5 to 15, *p* < 0.001). These preliminary results warrant further confirmation in larger, adequately powered longitudinal cohorts. Future studies should determine whether BCPs provide additional value beyond established nutritional screening and individual BC parameters in clinical practice.

## Simple Summary

By applying unsupervised machine learning techniques, this cross-sectional study proposed a novel approach to simplify the clinical interpretation of body composition in newly diagnosed patients with gastrointestinal cancers, a population at high risk of malnutrition. To evaluate the relevance of this approach, we explored the association between the identified body composition profiles, nutritional risk, and health-related quality of life. Our findings suggest that male patients with Low Muscle Profile had a three-fold higher risk of malnutrition compared to those with High Muscle Profile. Nevertheless, malnutrition risk emerged as the only determinant of poorer quality of life, reflecting the importance of monitoring nutritional status throughout the cancer continuum. Further prospective longitudinal studies in larger cohorts are needed to evaluate the robustness and reproducibility of body composition profiles, while accounting for cancer site heterogeneity, disease-specific characteristics and clinical factors. Such studies should also clarify their added value beyond traditional, validated body composition measures routinely used in clinical practice.

## 1. Introduction

Gastrointestinal (GI) cancers comprise a heterogeneous group of malignancies affecting the digestive tract and associated abdominal organs, including the esophagus, stomach, colon and rectum, liver, and pancreas. In 2022, these cancers accounted for an estimated 4,783,391 new cases and 3,235,719 deaths worldwide [1]. Cancer-related malnutrition is a condition frequently observed among patients with GI malignancies: its prevalence ranges from 13% to 97% [2], depending also on diagnostic criteria, treatment stage and tumor site [3,4]. Specifically, the highest prevalence has been observed in pancreatic cancer (51%), followed by upper GI cancers (40%) and colorectal cancer (30%) [4]. In these patients, nutritional status alterations can occur from early phases of disease: indeed, GI cancers may impair the physiological feeding process as well as affect central regulation systems of satiety and hunger. As a consequence, these alterations may compromise the various phases of nutritional intake, digestion and absorption. In addition, adverse effects of anticancer treatments that include chemotherapy, radiotherapy and major surgery may further exacerbate an already compromised nutritional status by inducing nutrition impact symptoms (NIS) such as diarrhea, constipation, abdominal bloating, nausea and vomiting [5]. This clinical profile leads to muscle mass loss, decreased physical performance, and reduced Health-Related Quality of Life (HRQoL) [5], underscoring the need for a prompt and accurate assessment of malnutrition risk and timely intervention throughout the patient’s entire diagnostic and therapeutic pathway [3,6]. Such an approach is essential, as malnutrition is consistently associated with poorer clinical outcomes, including reduced treatment tolerance, increased therapy-related toxicity and higher risk of postoperative complications, longer hospital stays and increased healthcare costs [7]. On the other hand, sarcopenia, the progressive loss of skeletal muscle mass, muscle strength and physical performance, has emerged as significant prognostic factor in patients with GI cancers, being associated with an increased risk of post-surgical complications and reduced overall survival [8].

In this context, tailored nutritional interventions have demonstrated beneficial effects on nutritional status, quality of life, and psychological well-being in patients with GI cancers [9]. Nonetheless, traditional approaches based on nutritional risk screening and anthropometric measures may not comprehensively capture the complex alterations in nutritional status occurring over the course of the disease. Therefore, comprehensive nutritional assessment should incorporate the evaluation of body composition (BC), particularly muscle mass and skeletal muscle mass (SMM), which represents a key component in the diagnosis and management of malnutrition and sarcopenia [10,11]. Growing evidence indicates that cancer-related alterations in BC are associated with poorer treatment response, increased treatment-related toxicities, reduced HRQoL, and higher mortality risk [12,13]. In this regard, the assessment of muscle mass and physical performance may provide clinically relevant information beyond conventional measures such as body mass index (BMI), enabling the identification of potentially modifiable nutritional and functional impairments. At the same time, growing evidence highlights the importance of integrating nutritional assessment with composite indicators, such as the Geriatric Nutritional Risk Index, the Prognostic Nutritional Index, and the C-Reactive Protein-to-albumin ratio, which capture both nutritional reserves and systemic inflammation. Accordingly, emerging evidence suggests that nutritional status assessed through these indices may predict not only the risk of postoperative complications in patients with resectable pancreatic cancer but also the ability to tolerate and complete adjuvant therapy across commonly used chemotherapy regimens [14]. Moreover, low serum albumin levels and poorer performance status have been identified as independent and strong predictors of shorter survival in patients with pancreatic cancer and other gastrointestinal malignancies who are candidates for later-line systemic therapy, underscoring the clinical relevance of nutritional and inflammatory parameters in informing therapeutic decision-making, including in the advanced and end-of-life settings [15].

Furthermore, assessment of sarcopenia through imaging-based approaches may improve risk stratification and support the implementation of targeted interventions, such as nutritional optimization and personalized exercise programs [16,17].

Traditionally, computed tomography (CT) and magnetic resonance imaging (MRI) are considered gold-standard techniques for measuring SMM and skeletal muscle density (SMD) [18]. However, their use in routine assessment of BC is associated with higher costs, limited feasibility, and higher degree of invasiveness [13]. Bioelectrical impedance analysis (BIA) represents a widely used method for BC assessment in clinical nutrition practice due to its ease of use, portability, rapid execution, relatively low cost, and non-invasive nature. BIA estimates total body water (TBW) by measuring impedance (Z), which reflects the combination of resistance (R) and reactance (Xc). R represents the opposition to the flow of a weak alternating current, whereas Xc reflects the capacitive properties of cell membranes. Based on R values, BIA allows estimation of BC according to a two-compartment model, including fat mass (FM) and fat-free mass (FFM), through empirically derived equations [19].

In patients with GI cancers, growing evidence supports the utility of BIA-derived indices for assessing nutritional status in association with clinical outcomes. For instance, Scislo et al. demonstrated that BIA can identify differences in muscle mass across various GI cancer types and according to surgical intent (curative vs. palliative) [20]. Moreover, lower phase angle (PhA), an indicator of cellular integrity and hydration status, has been associated with a worse prognosis in patients with GI, including higher risk of postoperative infectious complications [21], poorer survival, reduced HRQoL and impaired physical condition, particularly in advanced stage of disease, supporting its prognostic value and its close association with nutritional indicators [22]. Consequently, the use of BIA enables a comprehensive and holistic evaluation of the patient’s health status, hydration balance, and cellular integrity by combining patient-reported data (such as: weight loss, reduced food intake, the presence of NIS), clinical assessments, and dietary data [22]. However, the large volume of data generated by BIA may complicate its interpretation in clinical practice [23]. To address this hurdle, a recent analysis proposed an unsupervised machine learning approach based on principal component analysis (PCA) and k-means clustering to synthetize BIA-derived indices through the identification of BC profiles (BCPs) in cancer patients at their first nutritional visit. This approach may facilitate the identification of patients at risk of malnutrition and sarcopenia and provide a better understanding of the association between BCPs and HRQoL [24]. Accordingly, this study aimed to identify, in a clinically relevant population of patients with GI cancers, BCPs derived from BIA indices using the same statistical approach. This method may offer the opportunity to enhance the interpretation of BIA-derived indices in clinical practice and potentially support patient nutritional risk stratification at the first nutritional assessment. Following identification of these profiles, we further aimed to preliminarily explore their association with malnutrition risk and HRQoL, assessed by the comprehensive Summary Score (SumSc) and by the overall Symptoms and Functional scores.

## 2. Materials and Methods

### 2.1. Study Design

This single-center, cross-sectional analysis includes newly diagnosed patients with GI cancers enrolled in the NUTRItional and sarcopenia SCREENing (NUTRISCREEN) project which is, in turn, part of the ONCOCAMP, a retrospective–prospective observational study (ClinicalTrials.gov ID: NCT06270602 on 21 February 2024) conducted within the Campania Oncology Network (Rete Oncologica Campana, ROC) [25]. To date, the NUTRISCREEN was primarily implemented at the Istituto Nazionale Tumori IRCCS “Fondazione G. Pascale” of Naples and involves multiple health professional figures, including clinical nutrition biologists and dietitians, physicians, statisticians, pharmacists, researchers and case managers, following a multidisciplinary and integrated approach (Appendix A). The primary aim of the NUTRISCREEN is to evaluate the nutritional status of cancer patients during the intake and management phase by the Multidisciplinary Oncology Groups. Further details on this nutritional project have been described elsewhere [24,26]. This study was conducted in accordance with the Declaration of Helsinki and approved by the Ethics Committee of the Istituto Nazionale Tumori IRCCS “Fondazione G. Pascale” of Naples (registration number 6/23 OSS “ONCOCAMP” on 10 March 2023).

### 2.2. Data Collection

For this analysis, data were collected from newly diagnosed patients with GI cancers enrolled in the NUTRISCREEN project between July 2023 and July 2025. All data were obtained during the first nutritional visit at our cancer center using a Case Report Form designed by study staff. We included patients diagnosed with stomach, pancreas, rectum cancers and hepatocellular carcinoma. Patients with missing data on nutritional risk were excluded. The following socio-demographic, lifestyle and clinical variables were collected at time of the first nutritional consultation and included in this analysis: gender (male or female), age (years), education level (primary school, middle school, high school, and university degree or higher), smoking status (never smoker, former smoker, or current smoker), presence of intermediate risk factors or comorbidities (specifically: hypertension, dyslipidemia and type 2 Diabetes).

### 2.3. Assessment of Malnutrition Risk

The risk of malnutrition has been assessed using the Nutritional Risk Screening 2002 (NRS-2002) [27]. This validated screening tool identifies patients at risk of malnutrition in hospital settings by evaluating two components: nutritional status (defined based on BMI, weight loss over the preceding three months, and reduced dietary intake during the preceding week) and disease severity (reflecting the impact of acute or chronic illness on nutritional requirements). The NRS-2002 scoring system ranges from 0 to 7, with a score of ≥3 reflecting a higher risk of malnutrition and the need for tailored nutritional intervention [27]. The risk of sarcopenia has been assessed using the Strength, Assistance with walking, Rising from a chair, Climbing stairs, and Falls (SARC-F) questionnaire [28]. This validated screening tool is widely used to identify patients with suggestive signs of sarcopenia. It includes five questions assessing muscle strength, assistance with walking, rising from a chair and falls, each with three possible responses except for the question regarding falls: “No difficulty”, “Some difficulty”, or “Severe difficulty or inability to perform the task”. Falls-related responses are classified into three categories: “None,” “1–3 falls,” and “4 or more falls”. For each of these responses, a score of 0, 1, or 2, was assigned, respectively. The total score ranges from 0 to 10, with higher scores reflecting a higher risk of sarcopenia. Specifically, a score of 4 or higher is typically considered indicative of sarcopenia risk, requiring further diagnostic evaluation and potential intervention [28].

### 2.4. Anthropometric Measurements and BC Analysis

Anthropometric measurements, including weight, height, and waist and hip circumferences (in centimeters, cm) were collected. Body weight and height were measured using a calibrated mobile scale equipped with a stadiometer (WUNDER C201, Wunder Sa.Bi. srl, Milan, Italy) while circumferences were assessed using a non-elastic measuring tape (SECA 201; SECA, Hamburg, Germany). BMI was calculated using the formula weight (kg)/height (m^2^) and classified according to the World Health Organization (WHO) recommendations [29]. BC was assessed using BIA with an Akern 101 bioimpedance device (version 3.4.28; Akern Srl, Pontassieve, Italy) at a frequency of 50 Hz under standardized conditions. This analysis was performed in the morning, with patients in a fasting state to ensure greater measurement accuracy. Patients were asked to lay in a supine position. Subsequently, a trained nutritionist from the study staff positioned the electrodes at four standardized anatomical sites on the hands and feet.

Among BIA-derived parameters, PhA (expressed in degrees, °) was directly calculated as the arctangent of the ratio between Xc and R. Moreover, BIA provides the estimation of FM, FFM, body cell mass (BCM), SMM, appendicular skeletal muscle mass (ASMM), extracellular water (ECW), intracellular water (ICW), and the ECW/ICW ratio. These parameters were expressed in kilograms (kg), except for ECW, ICW (expressed in liters, L) and ECW/ICW ratio. Moreover, relative indices of muscle and fat mass were calculated by normalizing the respective parameters to the square of the individual’s height (expressed in m^2^), including the Fat Mass Index (FMI), Fat-free Mass Index (FFMI), Body Cell Mass Index (BCMI), Skeletal Muscle Index (SMI), and Appendicular Skeletal Muscle Index (ASMI).

### 2.5. HRQoL Assessment

HRQoL was evaluated using the European Organization for Research and Treatment of Cancer core Quality of Life Questionnaire (EORTC QLQ-C30, version 3.0) [30], a validated instrument specifically developed to assess physical, psychological, and social functioning in patients with cancer. The questionnaire consists of 30 items and includes five functional scales (physical, role, emotional, cognitive, and social functioning), three symptom scales (fatigue, nausea and vomiting, and pain), six individual items assessing additional symptoms (dyspnea, insomnia, appetite loss, constipation, diarrhea, and financial difficulties), and a global health status/quality of life scale (GHS). The latter is derived from the mean of two items assessing patients’ perception of their overall health and quality of life during the preceding week.

Responses are rated on a four-point Likert scale, ranging from 1 (“Not at all”) to 4 (“Very much”), whereas the GHS/quality of life scale is scored on a seven-point scale, ranging from 1 (“Very poor”) to 7 (“Excellent”). All scores are linearly converted to a 0–100 scale. Higher scores on the functional scales and GHS reflect better functioning and quality of life, while higher scores on the symptom scales indicate a greater symptom burden.

The EORTC QLQ-C30 SumSc was derived by averaging 13 QLQ-C30 scales, with the GHS and financial impact item excluded from the calculation. Before computing the SumSc, symptom scale scores were reverse-coded to ensure a consistent direction of interpretation across all scales. The SumSc was calculated only when data were available for all 13 scales, following the *EORTC QLQ-C30 Scoring Manual*, with individual scale scores calculated when at least 50% of the corresponding items had been completed [31]. To provide a single composite indicator of overall functioning and symptom burden, the mean scores of the functional and symptom scales were calculated and subsequently defined as the overall Functional Score and overall Symptom Score, respectively.

### 2.6. Statistical Analysis

Categorical variables were summarized as absolute frequencies and percentages, while continuous variables were presented as mean ± standard deviation (SD). Potential associations between malnutrition risk, anthropometric measurements, BIA-derived indices and HRQoL were evaluated through univariate analyses. Associations between categorical variables were evaluated using the Chi-squared test, while the Wilcoxon rank-sum test was used for continuous variables. To account for multiple comparisons, the Benjamini–Hochberg procedure was applied to control the false discovery rate (FDR) [31]. Subsequently, Bartlett’s test of sphericity was significant (χ^2^ = 1932.83, *p* < 0.001), indicating that the correlation matrix significantly differed from an identity matrix [32]. Later on, k-means clustering [33] algorithm was performed on the principal component scores obtained by assessing PCA on the six BIA-derived BC indices, rather than on the raw standardized variables. PCA is a dimensionality-reduction technique that transforms a set of correlated continuous variables into a smaller number of uncorrelated components while preserving as much of the original variance as possible. BIA-derived indices were automatically standardized prior to PCA. The Kaiser criterion [34] was adopted to retain the first two Principal Components (PCs). The factor loadings were displayed to evaluate the single PCs collecting correlation patterns given the input variables. The k-means algorithm is an unsupervised machine learning method for clustering that groups observations in Euclidean space, maximizing similarity within each cluster and minimizing similarity between clusters. The suitability of the dataset for clustering was assessed using the Hopkins statistic [35]. The optimal number of clusters was two according to the Silhouette plot method. To formally assess the stability and reproducibility of the two-clusters solution, we performed a bootstrap resampling validation using the Jaccard similarity method of Hennig [36] with 1000 resamples. Both clusters demonstrated high stability (mean Jaccard similarity = 0.94 for LMP and 0.94 for HMP; values higher than 0.85 conventionally considered as the cut-off referred to high stability). After assessing the optimal number of clusters, multivariable logistic regression models, adjusted for age, gender, cancer type, educational level and BMI were used to evaluate the association between nutritional risk and BC clusters. Additionally, linear regression models adjusted for age, gender, education levels and cancer type were fitted to examine the relationships among HRQoL scores (SumSc, Functional Score, Symptoms Score) and patients’ nutritional characteristics. Moreover, a multivariable analysis examining the association between malnutrition risk and BCPs was further stratified by gender. All statistical tests were two-sided, at a 95% significant confidence level. Statistical analyses were performed using R statistical software (version 4.5.2; R Core Team, R Foundation for Statistical Computing, Vienna, Austria, 2025). The following packages and versions were employed: dplyr (1.2.1), tidyr (1.3.2), purrr (1.2.2), FactoMineR (2.15), factoextra (2.0.0), EFAtools (0.8.0), hopkins (1.1) and FactoMineR (2.15).

## 3. Results

### 3.1. Descriptive Statistics

One-hundred eighty-nine patients with GI cancers, with a mean age of 66.3 ± 11.2 years at the first nutritional visit were included (Table 1). The most prevalent cancer type was gastric (37%), followed by pancreatic (26.5%) and rectal cancers (24.3%), while 12.2% of patients had a diagnosis of hepatocellular carcinoma. More than half (65.1%) of participants were male, 81.5% engaged/cohabitating and 45% former smokers. Regarding education, 52.1% of participants had completed middle school or lower, 31.4% completed high school and 16.5% held a university degree or higher. Hypertension and dyslipidemia were the most prevalent intermediate risk factors observed in this group of patients, with corresponding percentages of 45.0% and 25.9%, respectively. The analysis of nutritional risk indicated that about 36.5% of patients were at risk of malnutrition (NRS-2002 ≥ 3) and 32.3% were at risk of sarcopenia (SARC-F ≥ 4). Regarding general nutritional status, defined according to BMI categories, about 43% of patients were normal-weight, 38% overweight and 19% obese. Overall, mean waist circumference was 92.6 ± 10.8 cm. BC analysis, assessed by BIA, showed a mean PhA of 5.8 ± 1.2°, while the mean ECW/TBW and ICW/TBW ratios were 0.5 ± 0.1 and 0.5 ± 0.1, respectively. Mean SumSc was 75.6 ± 16.3. Patients also reported a mean Functional Score of 75.2 ± 18.4 and a Symptom Score equal to 24.0 ± 16.6.

### 3.2. Patient’s Characteristics According to Malnutrition Risk

Table 2 presents the descriptive characteristics of participants according to malnutrition risk, as evaluated by the NRS-2002. Patients at risk of malnutrition (NRS-2002 ≥ 3) were significantly older compared to those not at nutritional risk (71.9 ± 9.6 vs. 63.1 ± 10.9 years, respectively; *p* < 0.001). Statistically significant differences (*p* < 0.001) were also observed according to cancer type. Patients with gastric cancer were more likely to be at nutritional risk than those with other cancer types (58.0% vs. 25.0%), whereas patients with rectal cancer were less frequently at nutritional risk (5.8% vs. 35.0%; *p* < 0.001). Nutritional risk did not differ significantly according to gender or smoking habit; however, statistically significant differences were observed according to educational level (*p* = 0.011). Moreover, patients at risk had also an increased prevalence of intermediate risk factors and comorbidities, namely hypertension and type 2 Diabetes compared to those not at nutritional risk (62.3% vs. 36.7%, *p* = 0.004 for hypertension and 30.4% vs. 15.8%, *p* = 0.054, for type 2 Diabetes). Patients at nutritional risk were more frequently at risk of sarcopenia and had a lower BMI compared to those not at nutritional risk (56.5% vs. 18.3%, *p* < 0.001 for sarcopenia risk; 25.1 ± 4.4 vs. 26.6 ± 4.2 kg/m^2^ for BMI). Regarding BC estimated using BIA, patients at risk of malnutrition had lower values of PhA compared to not at risk (5.6 ± 1.5 vs. 5.9 ± 1°, *p* = 0.006), basal metabolic rate (BMR, 1516.4 ± 198.5 vs. 1614.0 ± 205 kcal/day, *p* = 0.003), BCM (26.4 ± 6.8 vs. 29.8 ± 7.1 kg, *p* = 0.003), FM (15.2 ± 8.5 vs. 18.0 kg, *p* = 0.010), FFM (51.8 ± 8.9 vs. 56.0 ± 10.7 kg, *p* = 0.013) and ASMM (19.2 ± 4.0 vs. 21.3 ± 4.7, *p* = 0.005). Malnutrition risk was also correlated with reduced hydration status compared to patients not at risk, such as TBW (38.6 ± 7.0 vs. 41.3 ± 8.0 L, *p* = 0.021), ICW (19.8 ± 5.0 vs. 22.2 ± 5.1 L, *p* = 0.003), ECW/ICW (1.0 ± 0.3 vs. 0.9 ± 0.2, *p* = 0.006) and ICW/TBW (0.5 ± 0.1 vs. 0.5 ± 0.0, *p* = 0.041). Finally, compared with patients not at risk, those at nutritional risk reported lower SumSc (70.4 ± 16.9 vs. 78.7 ± 15.2, *p* < 0.001), lower Functional Score (70.9 ± 19.7 vs. 77.6 ± 17.2, *p* = 0.021), and higher Symptoms Score (29.6 ± 16.7 vs. 20.8 ± 15.7, *p* < 0.001).

### 3.3. Identification of BCPs Through PCA and k-Means Clustering

**Figure 1:**
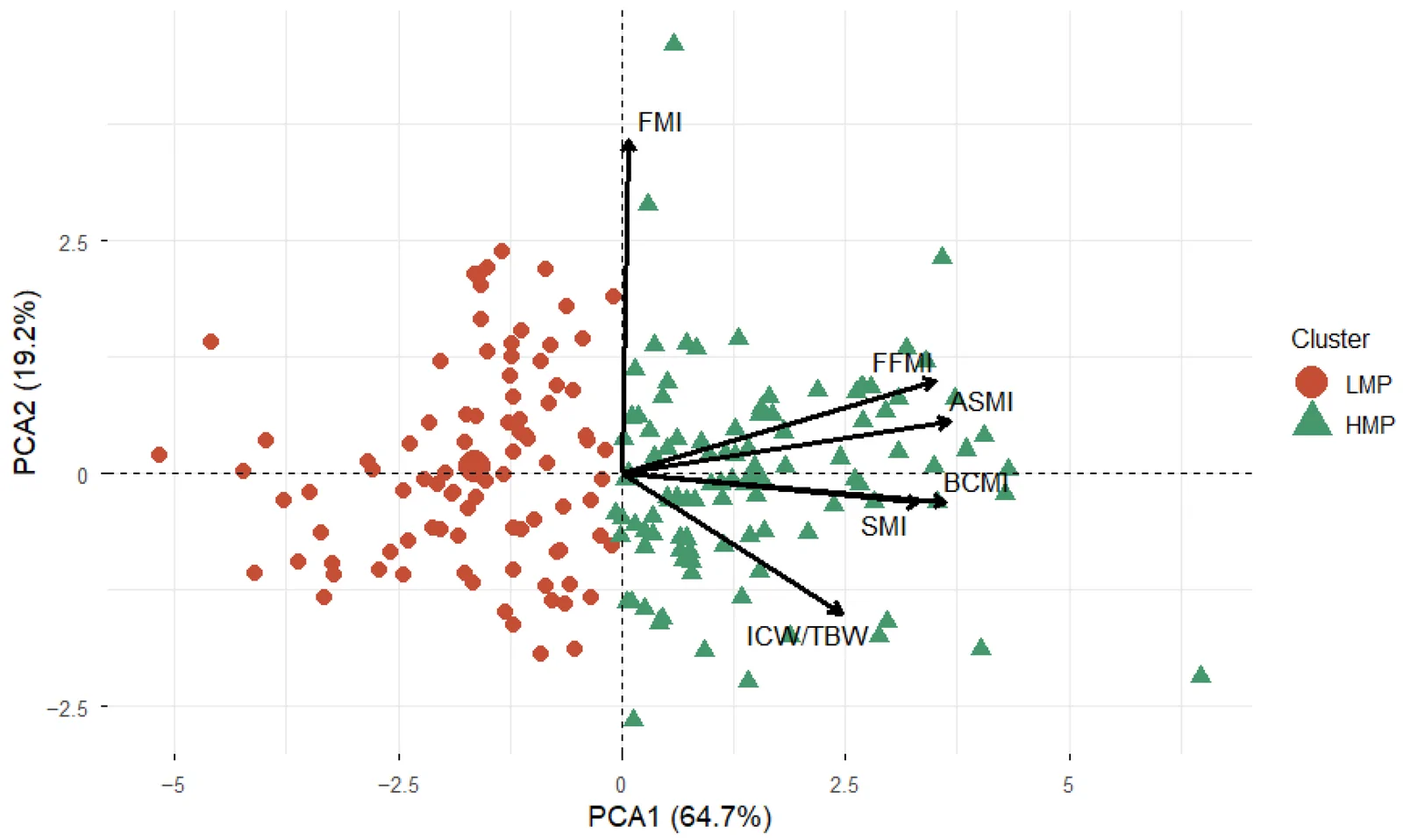
PCA results and k-means clustering outputs.

PCA identified two dominant components: PC1, which explained 64.7% of the total variance, and PC2, which explained an additional 19.2% (Figure 1). Further dimensions were not retained, as all remaining components showed eigenvalues < 1. PC1 was largely influenced by BC parameters related to muscle and cellular mass, with high factor loadings observed for ASMI (0.97), BCMI (0.95), FFMI (0.92), and SMI (0.87). In contrast, PC2 was mainly characterized by fluid distribution and fat mass indicators, showing a strong positive loading for FMI (0.94) and a moderate negative loading for the ICW/TBW ratio (−0.40). The Hopkins statistic (H = 0.99, *p* < 0.001) indicated a strong tendency toward cluster formation, supporting the suitability of the dataset for clustering analysis. K-means clustering identified two groups, namely Low Muscle Profile (LMP, n = 90) and High Muscle Profile (HMP, n = 99). The silhouette method indicated a maximum average width of 0.34 at k = 2 (Figure S1). These clusters were analyzed regarding to gender, nutritional and sarcopenia risks, quality of life and BC characteristics, in particular, muscle mass, body cellular mass and integrity, and fluid balance.

### 3.4. Patient’s Characteristics According to BCPs

As shown in Table 3, where patients’ characteristics are described according to BCPs, no statistically significant differences were found between HMP and LMP regarding age or cancer types. LMP was most frequently represented by female patients compared to HMP (63.3% vs. 9.1%, *p* < 0.001). Moreover, patients in LMP were more frequently at risk of malnutrition and sarcopenia compared to HMP (47.8% vs. 26.3%, *p* = 0.004 for malnutrition risk and 44.4% vs. 21.2%, *p* = 0.002 for sarcopenia risk). Statistically significant differences were also observed for body weight, BMI, waist and hip circumferences (all *p*-value < 0.001). Notably, statistically significant differences were observed between LMP and HMP across BMI categories: specifically, patients with LMP were more frequently normal-weight compared patients with HMP (58.9% vs. 29.3%, *p* < 0.001). All BIA-derived indices were individually correlated with BCPs, except for Xc, FM and FMI (for all values, *p* < 0.001). Finally, patients in LMP had poorer SumSc (72.3 ± 16.6 vs. 78.7 ± 15.4, *p* = 0.005), Functional Score (71.2 ± 19.3 vs. 78.7 ± 16.9, *p* = 0.007) and higher Symptom Score (26.9 ± 16.8 vs. 21.4 ± 16.0, *p* = 0.022) compared to those in HMP.

### 3.5. Malnutrition Risk and Patients’ Characteristics

Table 4 presents the results of the multivariate analysis performed to evaluate the association between BCPs and malnutrition risk in the entire cohort. We also analyzed the total number of patients at risk of malnutrition relative to the total number of patients within each BCP. Accordingly, 26.3% and 47.8% of patients were at risk of malnutrition (NRS-2002 ≥ 3) in HMP and LMP, respectively. Multivariable analysis suggests that patients in LMP had a two-fold, although not significant, increased risk of malnutrition compared to those with HMP (OR: 2.07, 95% CI: 0.74–6.04, *p* = 0.171).

Table 5 presents the results of the multivariable analysis stratified by gender, evaluating the association between BCPs and malnutrition risk. In the HMP group, 25.6% of male patients and 33.3% of female patients were at risk of malnutrition. In the LMP group, nutritional risk was identified in 72.7% of male patients and 33.3% of female patients. The gender-stratified analysis adjusted for age, cancer type, educational level and BMI suggested a potential, although marginally significant association between the LMP and malnutrition risk among male patients, with those in the LMP showing approximately three-fold higher risk of malnutrition compared with those in the HMP (OR = 3.77, 95% CI: 1.08–14.3, *p* = 0.042). Conversely, in female patients, BCPs were not associated with an increased risk of malnutrition (OR = 0.33, 95%CI: 0.03, 3.06, *p* = 0.331).

### 3.6. Association Between SumSc, Symptom Score, Functional Score and Patients’ Nutritional Characteristics

**Figure 2:**
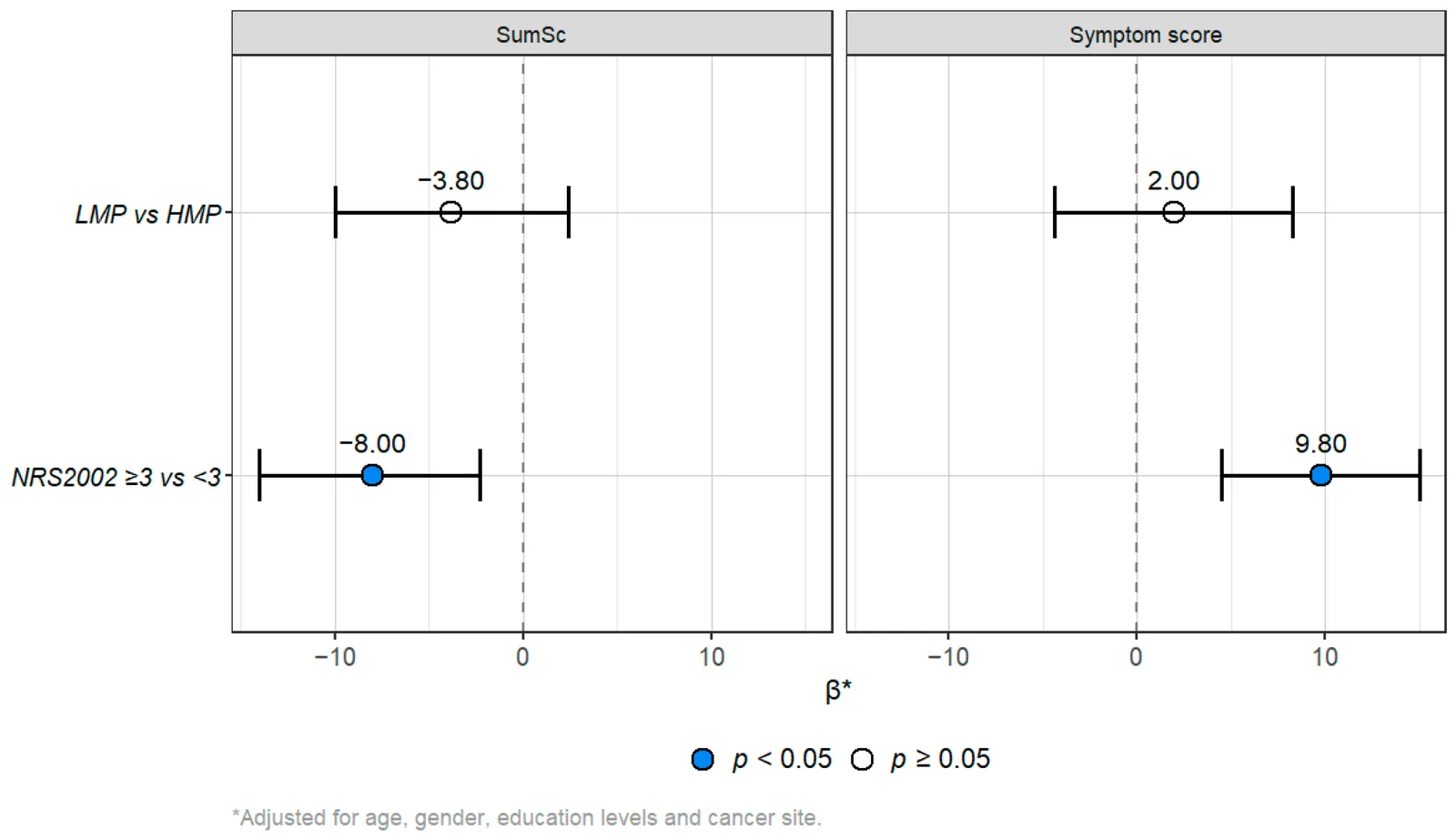
Association between SumSc, Symptom Score and nutrition-related factors.

When we investigated the association between SumSc and patients’ nutritional characteristics, at multivariable analysis adjusted for age, gender, education level and cancer type, malnutrition risk emerged as the only determinant significantly associated with poorer HRQoL. Specifically, patients at nutritional risk exhibited a mean SumSc that was approximately 8 points lower than that observed in patients not at nutritional risk (β = −8.0, 95% CI: −14 to −2.3, *p* = 0.007). BCPs were not associated with SumSc (Figure 2). Similarly, patients at nutritional risk reported a significantly higher Symptom Score compared to those not at risk (β = 9.8, 95% CI: 4.5 to 15, *p* < 0.001) (Figure 2). No significant associations were observed between overall Functional Score and patients’ nutritional risk (β = −4.7, 95% CI: −12 to 2.8, *p* = 0.2).

## 4. Discussion

In this cross-sectional analysis, we proposed an original approach based on PCA and k-means clustering to integrate the large amount of data generated by BIA and identify BCPs in newly diagnosed patients with GI cancers at their first nutritional visit. Moreover, to evaluate the clinical utility of BCPs in this group of patients, we preliminarily explored their association with malnutrition risk and HRQoL. Accordingly, in our analysis, we derived two profiles, namely, HMP and LMP. Statistically significant differences were observed between these two clusters across BMI categories: specifically, patients with LMP were more frequently normal-weight compared to patients with HMP. Conversely, patients with HMP were more frequently overweight or obese and exhibited higher waist and hip circumferences. Moreover, the HMP group was predominantly composed of male patients, whereas the LMP group was more frequently represented by female patients.

When we evaluated the association between BCPs and nutritional risk at multivariable analysis, no significant association were observed in the entire cohort; at multivariate analysis stratified by gender, our results suggest that male patients in the LMP group had an increased risk of malnutrition. A substantial body of evidence indicates that gender-specific factors can significantly influence the nutritional status and outcomes in cancer patients [37]. Accordingly, men typically have a higher proportion of FFM, whereas women exhibit a relatively greater FM, which may influence their response to cancer-related malnutrition and treatment. In this regard, Yoon et al. observed gender-specific differences in BC of patients with GI cancers. Firstly, female patients had lower value of PhA compared to male patients. Secondly, male patients had significantly lower ICW and higher FFM compared to those of female patients. The authors suggest that the latter differences were particularly relevant, as the coexistence of lower ICW and higher FFM may indicate a more pronounced inflammation [38]. Similarly, Al-Bayyari et al. found that male patients with GI cancers showed greater muscle mass depletion (as assessed by BIA-derived FFMI) compared to female patients, although no significant differences were observed in overall malnutrition risk. When stratified by cancer stage, male patients with advanced disease showed a significantly higher risk of malnutrition, weight loss and prevalence of acute disease. Furthermore, inadequate dietary intake was associated with increased malnutrition risk and muscle mass depletion in both genders. However, only male patients with reduced food intake reported poorer functional scale outcomes, whereas this association was not observed in female patients [37]. Although our preliminary findings may suggest a potential association between male sex within the LMP and nutritional risk, this result should be interpreted with caution. Our aim was not to emphasize the role or relevance of gender per se, but rather to describe the potential patterns emerging from the association between the identified BCPs and nutritional risk.

In this context, the use of BIA in clinical practice, combined with the evaluation of dietary intake, may support the longitudinal monitoring of changes in BC and dietary intake in both genders, thereby facilitating the implementation of personalized nutritional interventions [39]. Future studies should also aim to clarify the underlying mechanism driving the latter differences observed [40].

The impact of BC on cancer prognosis is increasingly recognized. Its growing relevance in the scientific literature reflects the need to identify novel host-related prognostic markers that may help explain the variability in clinical outcomes in conjunction with well-established biomarkers, thereby enabling a more accurate and comprehensive assessment of individual patient risk [41]. Accordingly, sarcopenia has emerged as a significant prognostic factor associated with poorer survival outcomes and an increased risk of major postoperative complications [8] as well as higher rates of hospital readmission in patients with GI cancers [42]. The latter evidence underscores the importance of integrating preoperative assessment with appropriate tests for the diagnosis of sarcopenia, combining BC analysis with measures of muscle strength. This comprehensive approach may facilitate the early identification of patients at risk and enable the timely implementation of preventive and supportive interventions aimed at improving clinical outcomes.

Unlike sarcopenia, which primarily reflects a reduction in muscle mass and strength, myosteatosis is characterized by the abnormal accumulation of adipose tissue within skeletal muscle. This condition has emerged as a novel marker of impaired muscle quality and metabolic dysfunction, capturing qualitative changes in skeletal muscle composition associated with metabolic alterations, systemic inflammation, and insulin resistance. These pathophysiological mechanisms may impair treatment tolerance and potentially contribute to tumor progression. In this context, a recent meta-analysis of observational studies suggests that myosteatosis is associated with reduced overall survival in patients with esophageal and gastro-esophageal junction cancer, supporting its potential as prognostic marker [41]. Moreover, the compresence of myosteatosis and systemic inflammation, evaluated using the neutrophil-to-lymphocyte ratio, has been associated with poorer disease-free survival and overall survival in patients with resectable gastric cancer, suggesting that both these factors regularly evaluated in clinical practice may improve risk stratification in this patient population [43]. Similarly, Sahin et al. investigated the prognostic value of the albumin-myosteatosis gauge (AMG) on survival outcomes of patients with advanced pancreatic cancer treated with first-line chemotherapy, reflecting the combined role of impaired muscle quality and systemic inflammation. In this study, lower values of AMG were significant associated with poorer overall survival [44], emphasizing the need for further studies aiming at evaluating these findings. This novel index, combining the assessment of muscle quality and health with serum albumin as a marker of nutritional and inflammatory status, may have the potential for routine implementation in clinical practice, being based on laboratory and instrumental assessments that are readily accessible through patients’ medical records [45].

An essential aspect of our analysis pertains to the timing of nutritional status assessment. In the context of the NUTRISCREEN project, patients undergo a structured assessment, including nutritional risk evaluation, at the beginning of the diagnostic–therapeutic pathway. This implies that the first nutritional consultation is incorporated into and performed alongside the clinical, instrumental, and therapeutic assessments routinely required as part of patients’ care pathways. On the other hand, because nutritional assessment takes place at a relatively early stage of the care pathway, patients are, in most cases, still undergoing staging work-up and therapeutic planning. Consequently, information regarding disease characteristics and anticancer treatments, as well as hospitalization and other clinically relevant events may not yet be available or definitively established at the time of the first nutritional assessment. This further highlights the need for longitudinal studies that, beyond providing an initial assessment of nutritional status, can monitor its evolution over time and investigate its associations with anticancer treatments, clinical events and disease characteristics.

Accordingly, we observed that at first nutritional assessment, the prevalence of malnutrition risk (as evaluated by the NRS-2002) was 36.5% in the overall cohort, while the prevalence of patients at risk of sarcopenia was 32.3%. In the literature, a comparable proportion of patients at risk of malnutrition has been reported in the prospective cohort study (the PREMAS study) by Duran Poveda et al. [46]. Specifically, the prevalence of malnutrition risk among patients with a primary diagnosis of GI cancers at time of hospital admission for major surgery was 39%, the mean age of patients was 68.2 years, while mean BMI was 26.7 kg/m^2^, similar to our results (66.3 years and 26 kg/m^2^). In fact, both studies were conducted in Mediterranean populations, which may enhance the comparability of the results. Nevertheless, some key differences should be considered, as in the Mediterranean cohort by Duran Poveda et al., nutritional risk was assessed using a different tool, the Malnutrition Universal Screening Tool (MUST), and patients were evaluated in the perioperative setting (specifically within 48 h of hospital admission), whereas in our cohort the assessment was performed at the beginning of the diagnostic and therapeutic care pathway. This methodological difference may partly explain the observed variations in the prevalence of malnutrition risk, given that nutritional status is dynamic and may worsen over the course of disease progression and treatment [47]. Consequently, cross-sectional comparisons should be interpreted with caution.

In this analysis, we also observed that nutritional risk was higher in elderly patients and in those with hypertension and type 2 Diabetes. These findings suggest that frailer and more vulnerable patient groups tend to present a higher risk of malnutrition from the early phase of the care pathway [48]. These results are in line with the existing literature, indicating that older patients with GI cancers may be particularly vulnerable due to alterations in GI functions, which can contribute to vitamin D deficiency, osteoporosis [49], and an increased risk of sarcopenia. Notably, clinical risk in many older cancer patients may be exacerbated by the co-occurrence of malnutrition, sarcopenia, and frailty [50]. In this regard, evidence from a study involving adults aged ≥70 years with cancer, of whom 33% had upper GI cancers, showed that 83.3% presented with at least one of these conditions; 26.7% had only one condition, whereas 30.0% exhibited all three simultaneously [51].

As part of this cross-sectional analysis, we investigated the association between HRQoL and patients’ nutritional-related factors. Accordingly, higher malnutrition risk emerged as the sole determinant of poorer SumSc and higher Symptom Score, consistent with existing evidence in GI cancers [3,52,53,54]. Accordingly, evidence from a hospital-based, multi-center, cross-sectional study indicated that nausea, vomiting, loss of appetite, dysphagia, and the presence of at least three NIS were associated with moderate and severe malnutrition risk. Moreover, cancer site, particularly upper GI cancers, was identified as an independent predictor of increased nutritional risk, highlighting the importance of integrating the assessment of NIS into clinical practice [55]. Furthermore, evidence from a prospective, longitudinal survey involving 219 oncological patients found a high prevalence of NIS (79% and 72%, respectively) after one and six months from the beginning of chemotherapy, whilst the most common symptoms reported included dry mouth, constipation, nausea, diarrhea and stomach pain. The authors observed that higher number of symptoms correlated with poorer HRQoL and lower functional status [56]. These findings suggest that malnutrition risk is an independent predictor of poorer HRQoL in patients with GI cancers, potentially reflecting its close interrelationship with the development of NIS, which may further exacerbate a nutritional status impaired by the underlying malignancy and its treatment.

In our analysis, SumSc, Functional and Symptoms scores, were not associated with BCPs. Mechanistically, the association between reduced muscle mass and poorer HRQoL may be mediated by reductions in muscle strength and physical functioning [57]. However, declines in these parameters may occur over different timeframes, making it challenging to detect simultaneously both these alterations in cross-sectional studies [52]. In this regard, the EORTC QLQ-C30 assesses patients’ perceptions of their quality of life and experiences over the preceding week, whereas changes in BC and muscle mass may occur over a longer period. This highlights the need for prospective observational studies that monitor longitudinal changes in BC and evaluate their association with HRQoL domains in patients with GI cancers, while accounting for the potential factors that may influence nutritional status and quality of life, such as cancer stage and anticancer therapies. In this context, Hanna et al. suggested that low muscle mass at the time of diagnosis may not be directly associated with or influence HRQoL at baseline. However, when assessed longitudinally, progressive muscle loss over time appears to be more closely associated with this patient-reported outcome [52]. These results suggest the need for caution when interpreting the incremental value of a single cross-sectional assessment of BCPs in relation to a patient-reported outcome reflecting a relatively short and specific time window. Moreover, further studies are needed to evaluate the association between HRQoL and changes in BC also considering the impact of anticancer treatments on nutritional status.

This study has several limitations. First, its cross-sectional design precludes any causal inference regarding the observed associations. Therefore, the observed association between male patients in the LMP and increased risk of malnutrition should be interpreted with caution. Second, the absence of detailed disease- and treatment-related information, particularly cancer stage, oncological therapies, NIS, comorbidities, performance status, muscle strength and the date of cancer diagnosis, left potential confounding that limit the interpretation and generalizability of the findings. Moreover, the lack of information on cancer stage and anticancer therapies limited our ability to provide a clear, detailed, and comprehensive characterization of patients’ nutritional status and to perform sensitivity analysis. Similarly, another important limitation is the relatively small number of patients available for each tumor site, which precluded sensitivity analyses stratified by cancer site. This is particularly relevant given the clinical heterogeneity of selected GI malignancies in terms of nutritional impairment, disease biology, treatment pathways, and prevalence of cancer-related malnutrition. Further longitudinal studies including an appropriate number of patients for each tumor site are warranted to enable more robust statistical analyses and to better characterize potential differences in nutritional status and BC according to tumor location.

In addition, the single-center design and relatively small sample size may further reduce the external validity of the results. Therefore, our findings should be interpreted as exploratory and hypothesis-generating rather than conclusive. These limitations should be considered when interpreting the findings and underscore the need for larger, multi-center prospective studies specifically focused on patients with GI cancers, with an accurate collection of clinical and cancer-related data to confirm and extend our observations.

Despite these limitations, this study has also some points of strength. Using PCA and k-means clustering, we proposed an original approach to condense a large amount of data derived from BIA deriving two BCPs reflecting hydration status, cellular health, adiposity, and muscle mass. The integration of these profiles with socio-demographic, anthropometric and HRQoL measures provided a preliminary description of patients’ nutritional status. BCPs may therefore enhance and simplify clinical interpretation of BIA-derived parameters in GI cancers. Future prospective, longitudinal studies including larger cohorts of patients with GI malignancies with more comprehensive and detailed clinical information on comorbidities, disease stage, tumor characteristics, anticancer therapies and clinical outcomes are warranted. Such studies should aim to confirm the proposed statistical approach for identifying distinct BCPs and to assess their robustness, reproducibility and clinical relevance. Moreover, future research should investigate whether these integrated profiles provide additional value over individual BC parameters commonly used in clinical practice, such as the SMI, BCMI, ASMI and PhA. Furthermore, the association between BCPs, malnutrition risk and HRQoL should be further clarified.

## 5. Conclusions

In this cross-sectional analysis, we proposed an original approach to simplify the complexity of BIA-derived parameters through the identification of BCPs in newly diagnosed patients with GI cancers. By integrating data on hydration status, BCM, FM and cellular health, these profiles may potentially complement nutritional assessment of GI cancers in clinical practice. However, given the aforementioned limitations, our findings should be interpreted with caution and considered hypothesis-generating rather than definitive. Further prospective studies with a larger cohort and a more comprehensive data collection on clinical and disease characteristics are warranted to investigate the robustness, reproducibility and added value of the identified profiles compared with traditional BIA-derived parameters.

## Figures and Tables

**Table 1:** Descriptive statistics.

Variable	N = 189
Socio-demographic variables	
Age, mean (SD)	66.3 (11.2)
Gender, *n* (%)	
Female	66 (34.9%)
Male	123 (65.1%)
Civil status, (*n*, %)	
Bachelor	10 (5.3%)
Engaged/cohabitating	154 (81.5%)
Divorced	7 (3.7%)
Widow	18 (9.5%)
Education level, (*n*, %)	
Primary school	28 (14.9%)
Middle school	70 (37.2%)
High school	59 (31.4%)
University degree or higher	31 (16.5%)
Smoking habit, (*n*, %)	
Never smokers	47 (24.9%)
Former smokers	85 (45.0%)
Current smokers	57 (30.2%)
Clinical variables	
Cancer type, (*n*, %)	
Hepatocellular carcinoma	23 (12.2%)
Pancreatic cancer	50 (26.5%)
Rectal cancer	46 (24.3%)
Gastric cancer	70 (37.0%)
Hypertension, (*n*, %)	
No	102 (54.0%)
Yes	87 (46.0%)
Type 2 Diabetes, (*n*, %)	
No	149 (78.8%)
Yes	40 (21.2%)
Dyslipidemia, (*n*, %)	
No	140 (74.1%)
Yes	49 (25.9%)
NRS-2002, (*n*, %)	
<3	120 (63.5%)
≥3	69 (36.5%)
SARC-F, (*n*, %)	
<4	128 (67.7%)
≥4	61 (32.3%)
EORTC QLQ C-30, mean (SD)	
SumSc	75.6 (16.3)
Functional Score	75.2 (18.4)
Symptom Score	24.0 (16.6)
Anthropometric parameters	
Weight (Kg), mean (SD)	71.5 (14.2)
Waist circumference (cm), mean (SD)	92.6 (10.8)
Hip circumference (cm), mean (SD)	97.6 (11.5)
BMI (kg/m^2^), mean (SD)	26.0 (4.3)
BMI categories, (*n*, %)	
Normal-weight (<25 kg/m^2^)	82 (43.4%)
Overweight (25–30 kg/m^2^)	71 (37.6%)
Obese (≥30 kg/m^2^)	36 (19.0%)
Bioelectrical Impedance Analysis-derived parameters	
Resistance (ohm), mean (SD)	491.5 (73.5)
Reactance (ohm), mean (SD)	49.3 (11.4)
PhA (°), mean (SD)	5.8 (1.2)
BMR (kcal/day), mean (SD)	1578.4 (207.7)
BCM (kg), mean (SD)	28.6 (7.2)
FM (kg), mean (SD)	17.0 (8.1)
FFM (kg), mean (SD)	54.5 (10.3)
TBW (l), mean (SD)	40.3 (7.8)
ECW (l), mean (SD)	19.0 (3.7)
ICW (l), mean (SD)	21.3 (5.2)
ECW/ICW, mean (SD)	0.9 (0.2)
ECW/TBW, mean (SD)	0.5 (0.1)
ICW/TBW, mean (SD)	0.5 (0.1)
SMM (kg), mean (SD)	26.0 (6.5)
ASMM (kg), mean (SD)	20.5 (4.6)
BCMI (kg/m^2^), mean (SD)	10.3 (2.0)
FMI (kg/m^2^), mean (SD)	6.3 (3.2)
FFMI (kg/m^2^), mean (SD)	19.8 (2.4)
SMI (kg/m^2^), mean (SD)	9.4 (1.7)
ASMI (kg/m^2^), mean (SD)	7.4 (1.1)

Abbreviations: NRS-2002, Nutritional Risk Screening 2002; SARC-F, Strength, Assistance with walking, Rising from a chair, Climbing stairs, and Falls; BMI, Body Mass Index; PhA, Phase Angle; BMR, Basal Metabolic Rate; BCM, Body Cellular Mass; FM, Fat Mass; FFM, Fat-free Mass; TBW, Total Body Water; ECW, Extracellular Water; ICW, Intracellular Water; SMM, Skeletal Muscle Mass; ASMM, Appendicular Skeletal Muscle Mass; BCMI, Body Cell Mass Index; FMI, Fat Mass Index; FFMI, Fat-free Mass Index; SMI, Skeletal Muscle Index; ASMI, Appendicular Skeletal Muscle Index.

**Table 2:** Patients’ characteristics according to malnutrition risk (NRS-2002 ≥ 3).

Variable	NRS-2002 < 3 N = 120	NRS-2002 ≥ 3 N = 69	*p*-Value ^1,2^
Socio-demographic variables			
Age, mean (SD)	63.1 (10.9)	71.9 (9.6)	<0.001 ^2^
Gender, *n* (%)			0.593 ^2^
Female	44 (36.7%)	22 (31.9%)	
Male	76 (63.3%)	47 (68.1%)	
Civil status, (*n*, %)			0.560 ^2^
Bachelor	8 (6.7%)	2 (2.9%)	
Engaged/cohabitating	98 (81.7%)	56 (81.2%)	
Divorced	5 (4.2%)	2 (2.9%)	
Widow	9 (7.5%)	9 (13.0%)	
Education level, (*n*, %)			0.011 ^2^
Primary school	13 (10.9%)	15 (21.7%)	
Middle school	39 (32.8%)	31 (44.9%)	
High school	40 (33.6%)	19 (27.5%)	
University degree or higher	27 (22.7%)	4 (5.8%)	
Smoking habit, (*n*, %)			0.700 ^2^
Never smokers	32 (26.7%)	15 (21.7%)	
Former smokers	51 (42.5%)	34 (49.3%)	
Current smokers	37 (30.8%)	20 (29.0%)	
Clinical variables			
Cancer type, (*n*, %)			<0.001 ^2^
Hepatocellular carcinoma	16 (13.3%)	7 (10.1%)	
Pancreatic cancer	32 (26.7%)	18 (26.1%)	
Rectal cancer	42 (35.0%)	4 (5.8%)	
Gastric cancer	30 (25.0%)	40 (58.0%)	
Hypertension, (*n*, %)			0.004 ^2^
No	76 (63.3%)	26 (37.7%)	
Yes	44 (36.7%)	43 (62.3%)	
Type 2 Diabetes, (*n*, %)			0.054 ^2^
No	101 (84.2%)	48 (69.6%)	
Yes	19 (15.8%)	21 (30.4%)	
Dyslipidemia, (*n*, %)			0.732 ^2^
No	90 (75.0%)	50 (72.5%)	
Yes	30 (25.0%)	19 (27.5%)	
SARC-F, (*n*, %)			<0.001 ^2^
<4	98 (81.7%)	30 (43.5%)	
≥4	22 (18.3%)	39 (56.5%)	
EORTC QLQ C-30, mean (SD)			
SumSc	78.7 (15.2)	70.4 (16.9)	<0.001 ^1^
Functional Score	77.6 (17.2)	70.9 (19.7)	0.021 ^1^
Symptom Score	20.8 (15.7)	29.6 (16.7)	<0.001 ^1^
Anthropometric parameters			
Weight (Kg), mean (SD)	74.1 (14.3)	67.0 (13.0)	0.001 ^1^
Waist circumference (cm), mean (SD)	93.0 (11.5)	91.8 (9.5)	0.560 ^2^
Hip circumference (cm), mean (SD)	98.5 (11.8)	96.0 (10.9)	0.289 ^2^
BMI (kg/m^2^), mean (SD)	26.6 (4.2)	25.1 (4.4)	0.061 ^2^
BMI categories, (n, %)			0.102 ^1^
Normal-weight (<25 kg/m^2^)	45 (37.5%)	37 (53.6%)	
Overweight (25–30 kg/m^2^)	49 (40.8%)	22 (31.9%)	
Obese (≥30 kg/m^2^)	26 (21.7%)	10 (14.5%)	
Bioelectrical Impedance Analysis-derived parameters			
Resistance (ohm), mean (SD)	487.6 (73.4)	498.1 (73.8)	0.188 ^1^
Reactance (ohm), mean (SD)	50.2 (10.5)	47.9 (12.6)	0.050 ^1^
PhA (°), mean (SD)	5.9 (1.0)	5.6 (1.5)	0.006 ^1^
BMR (kcal/day), mean (SD)	1614.0 (205.2)	1516.4 (198.5)	0.003 ^1^
BCM (kg), mean (SD)	29.8 (7.1)	26.4 (6.8)	0.003 ^1^
FM (kg), mean (SD)	18.0 (7.7)	15.2 (8.5)	0.010 ^1^
FFM (kg), mean (SD)	56.0 (10.7)	51.8 (8.9)	0.013 ^1^
TBW (l), mean (SD)	41.3 (8.0)	38.6 (7.0)	0.021 ^1^
ECW (l), mean (SD)	19.2 (3.7)	18.7 (3.7)	0.391 ^1^
ICW (l), mean (SD)	22.2 (5.1)	19.8 (5.0)	0.003 ^1^
ECW/ICW, mean (SD)	0.9 (0.2)	1.0 (0.3)	0.006 ^1^
ECW/TBW, mean (SD)	0.5 (0.0)	0.5 (0.1)	0.006 ^1^
ICW/TBW, mean (SD)	0.5 (0.0)	0.5 (0.1)	0.041 ^2^
SMM (kg), mean (SD)	26.6 (6.6)	24.8 (6.0)	0.029 ^1^
ASMM (kg), mean (SD)	21.3 (4.7)	19.2 (4.0)	0.005 ^1^
BCMI (kg/m^2^), mean (SD)	10.6 (1.9)	9.8 (2.2)	0.041 ^2^
FMI (kg/m^2^), mean (SD)	6.6 (2.9)	5.8 (3.4)	0.186 ^2^
FFMI (kg/m^2^), mean (SD)	20.0 (2.5)	19.4 (2.2)	0.115 ^2^
SMI (kg/m^2^), mean (SD)	9.4 (1.6)	9.2 (1.7)	0.427 ^2^
ASMI (kg/m^2^), mean (SD)	7.6 (1.1)	7.2 (1.0)	0.041 ^2^

^1^ Wilcoxon rank-sum test; Fisher’s exact test; ^2^ Benjamini & Hochberg correction for multiple testing. Abbreviations: NRS-2002, Nutritional Risk Screening 2002; SARC-F, Strength, Assistance with walking, Rising from a chair, Climbing stairs, and Falls; BMI, Body Mass Index; PhA, Phase Angle; BMR, Basal Metabolic Rate; BCM, Body Cellular Mass; FM, Fat Mass; FFM, Fat-free Mass; TBW, Total Body Water; ECW, Extracellular Water; ICW, Intracellular Water; SMM, Skeletal Muscle Mass; ASMM, Appendicular Skeletal Muscle Mass; BCMI, Body Cell Mass Index; FMI, Fat Mass Index; FFMI, Fat-free Mass Index; SMI, Skeletal Muscle Index; ASMI, Appendicular Skeletal Muscle Index; EORTC QLQ C-30 SumSc, European Organization for Research and Treatment of Cancer Quality of Life Questionnaire C30 Summary Score.

**Table 3:** Patients’ characteristics according to BCPs.

Variable	LMP N = 90	HMP N = 99	*p*-Value ^1,2^
Socio-demographic variables			
Age, mean (SD)	68.0 (11.6)	64.8 (10.7)	0.081 ^2^
Gender, *n* (%)			<0.001 ^2^
Female	57 (63.3%)	9 (9.1%)	
Male	33 (36.7%)	90 (90.9%)	
Civil status, (*n*, %)			0.321 ^2^
Bachelor	3 (3.3%)	7 (7.1%)	
Engaged/cohabitating	72 (80.0%)	82 (82.8%)	
Divorced	3 (3.3%)	4 (4.0%)	
Widow	12 (13.3%)	6 (6.1%)	
Education level, (*n*, %)			0.435 ^2^
Primary school	17 (19.1%)	11 (11.1%)	
Middle school	34 (38.2%)	36 (36.4%)	
High school	25 (28.1%)	34 (34.3%)	
University degree or higher	27 (22.7%)	4 (5.8%)	
Smoking habit, (*n*, %)			0.474 ^2^
Never smokers	19 (21.1%)	28 (28.3%)	
Former smokers	44 (48.9%)	41 (41.4%)	
Current smokers	27 (30.0%)	30 (30.3%)	
Clinical variables			
Cancer type, (*n*, %)			0.246 ^2^
Hepatocellular carcinoma	7 (7.8%)	16 (16.2%)	
Pancreatic cancer	26 (28.9%)	24 (24.2%)	
Rectal cancer	19 (21.1%)	27 (27.3%)	
Gastric cancer	38 (42.2%)	32 (32.3%)	
Hypertension, (*n*, %)			0.310 ^2^
No	53 (58.9%)	49 (49.5%)	
Yes	37 (41.1%)	50 (50.5%)	
Type 2 Diabetes, (*n*, %)			0.335 ^2^
No	74 (82.2%)	75 (75.8%)	
Yes	16 (17.8%)	24 (24.2%)	
Dyslipidemia, (*n*, %)			0.249 ^2^
No	71 (78.9%)	69 (69.7%)	
Yes	19 (21.1%)	30 (30.3%)	
NRS-2002, (*n*, %)			0.004 ^2^
<3	47 (52.2%)	73 (73.7%)	
≥3	43 (47.8%)	26 (26.3%)	
SARC-F, (*n*, %)			0.002 ^2^
<4	50 (55.6%)	78 (78.8%)	
≥4	40 (44.4%)	21 (21.2%)	
EORTC QLQ C-30, mean (SD)			0.005 ^1^
SumSc	72.3 (16.6)	78.7 (15.4)	
Functional Score	71.2 (19.3)	78.7 (16.9)	0.007 ^1^
Symptom Score	26.9 (16.8)	21.4 (16.0)	0.022 ^1^
Anthropometric parameters			
Weight (Kg), mean (SD)	63.0 (10.8)	79.2 (12.5)	<0.001
Waist circumference (cm), mean (SD)	86.3 (9.5)	97.8 (9.0)	<0.001 ^2^
Hip circumference (cm), mean (SD)	92.2 (10.7)	102.1 (10.2)	<0.001 ^2^
BMI (kg/m^2^), mean (SD)	24.4 (4.0)	27.5 (4.0)	<0.001 ^2^
BMI categories, (n, %)			<0.001 ^1^
Normal-weight (<25 kg/m^2^)	53 (58.9%)	29 (29.3%)	
Overweight (25–30 kg/m^2^)	27 (30.0%)	44 (44.4%)	
Obese (≥30 kg/m^2^)	10 (11.1%)	26 (26.3%)	
Bioelectrical Impedance Analysis-derived parameters			
Resistance (ohm), mean (SD)	543.5 (60.8)	444.1 (47.6)	<0.001 ^1^
Reactance (ohm), mean (SD)	49.5 (11.5)	49.2 (11.3)	0.670 ^1^
PhA (°), mean (SD)	5.2 (0.9)	6.3 (1.2)	<0.001 ^1^
BMR (kcal/day), mean (SD)	1410.4 (114.5)	1731.1 (146.2)	<0.001 ^1^
BCM (kg), mean (SD)	22.8 (3.9)	33.8 (5.0)	<0.001 ^1^
FM (kg), mean (SD)	16.5 (7.8)	17.4 (8.3)	0.372 ^1^
FFM (kg), mean (SD)	46.5 (6.0)	61.8 (7.5)	<0.001 ^1^
TBW (l), mean (SD)	34.5 (4.9)	45.6 (5.9)	<0.001 ^1^
ECW (l), mean (SD)	17.4 (3.2)	20.5 (3.5)	<0.001 ^1^
ICW (l), mean (SD)	17.1 (2.8)	25.1 (3.6)	<0.001 ^1^
ECW/ICW, mean (SD)	1.0 (0.2)	0.8 (0.2)	<0.001 ^1^
ECW/TBW, mean (SD)	0.5 (0.1)	0.4 (0.0)	<0.001 ^1^
ICW/TBW, mean (SD)	0.5 (0.1)	0.6 (0.0)	<0.001 ^1^
SMM (kg), mean (SD)	21.1 (4.5)	30.4 (4.5)	<0.001 ^1^
ASMM (kg), mean (SD)	17.0 (2.9)	23.7 (3.3)	<0.001 ^1^
BCMI (kg/m^2^), mean (SD)	8.8 (1.2)	11.7 (1.5)	<0.001 ^2^
FMI (kg/m^2^), mean (SD)	6.5 (3.2)	6.1 (3.1)	0.441 ^2^
FFMI (kg/m^2^), mean (SD)	18.0 (1.4)	21.4 (1.8)	<0.001 ^2^
SMI (kg/m^2^), mean (SD)	8.1 (1.1)	10.5 (1.2)	<0.001 ^2^
ASMI (kg/m^2^), mean (SD)	6.5 (0.7)	8.2 (0.8)	<0.001 ^2^

^1^ Wilcoxon rank-sum test; Fisher’s exact test; ^2^ Benjamini & Hochberg correction for multiple testing. Abbreviations: BCPs, Body Composition Profiles; LMP, Low Muscle Profile; HMP, High Muscle Profile; NRS-2002, Nutritional Risk Screening 2002; SARC-F, Strength, Assistance with walking, Rising from a chair, Climbing stairs, and Falls; BMI, Body Mass Index; PhA, Phase Angle; BMR, Basal Metabolic Rate; BCM, Body Cellular Mass; FM, Fat Mass; FFM, Fat-free Mass; TBW, Total Body Water; ECW, Extracellular Water; ICW, Intracellular Water; SMM, Skeletal Muscle Mass; ASMM, Appendicular Skeletal Muscle Mass; BCMI, Body Cell Mass Index; FMI, Fat Mass Index; FFMI, Fat-free Mass Index; SMI, Skeletal Muscle Index; ASMI, Appendicular Skeletal Muscle Index.

**Table 4:** Association between malnutrition risk and BCPs in the entire cohort.

Variable	Overall			
	Events/N (%)	OR *	95% CI	*p*-Value
BCPs				
HMP	26/99 (26.3%)	—	—	—
LMP	43/90 (47.8%)	2.07	0.74, 6.04	0.171

* Adjusted for age, gender, cancer type, educational level and BMI; Abbreviations: CI = Confidence Interval, OR = Odds Ratio, BCPs = Body Composition Profiles, LMP = Low Muscle Profile, HMP = High Muscle Profile.

**Table 5:** Association between malnutrition risk and BCPs stratified by gender.

Variable	Male Cancer Patients (n = 123)				Female Cancer Patients (n = 66)			
	Events/N (%)	OR **	95% CI	*p*-Value	Events/N (%)	OR **	95% CI	*p*-Value
BCPs								
HMP	23/90 (25.6%)	—	—	—	3/9 (33.3%)	—	—	—
LMP	24/33 (72.7%)	3.77	1.08, 14.3	0.042	19/57 (33.3%)	0.33	0.03, 3.06	0.331

** Adjusted for age, cancer type, educational level and BMI; Abbreviations: CI = Confidence Interval, OR = Odds Ratio, BCPs = Body Composition Profiles, LMP = Low Muscle Profile, HMP = High Muscle Profile.

## Data Availability

The original data presented in the study are openly available in Zenodo at https://doi.org/10.5281/zenodo.21771547 (accessed on 3 August 2026).

## Supplementary Materials

The following supporting information can be downloaded at https://www.mdpi.com/article/10.3390/curroncol33100591/s1. Figure S1: Optimal number of clusters.

## Abbreviations

The following abbreviations are used in this manuscript:

GI cancers	Gastrointestinal cancers
NIS	Nutrition Impact Symptoms
HRQoL	Health-Related Quality of Life
BC	Body Composition
BMI	Body Mass Index
SMM	Skeletal Muscle Mass
CT	Computed Tomography
MRI	Magnetic Resonance Imaging
SMD	Skeletal Muscle Density
BIA	Bioelectrical Impedance Analysis
TBW	Total Body Water
Z	Impedance
R	Resistance
Xc	Reactance
FM	Fat Mass
FFM	Fat-free Mass
PhA	Phase Angle
BCPs	Body Composition Profiles
PCA	Principal Component Analysis
SumSc	Summary Score
ROC	Rete Oncologica Campana
NRS-2002	Nutritional Risk Screening 2002
SARC-F	Strength, Assistance with walking, Rising from a chair, Climbing stairs, and Falls
WHO	World Health Organization
BCM	Body Cell Mass
ASMM	Appendicular Skeletal Muscle Mass
ECW	Extracellular Water
ICW	Intracellular Water
FMI	Fat Mass Index
BCMI	Body Cell Mass Index
SMI	Skeletal Muscle Index
ASMI	Appendicular Skeletal Muscle Index
EORTC QLQ-C30	European Organization for Research and Treatment of Cancer core Quality of Life Questionnaire
GHS	Global Health Status
SD	Standard Deviation
FDR	False Discovery Rate
PC	Principal Component
BMR	Basal Metabolic Rate
LMP	Low Muscle Profile
HMP	High Muscle Profile
AMG	Albumin-myosteatosis Gauge
MUST	Malnutrition Universal Screening Tool

## Appendix A

The NUTRISCREEN Research Group includes: Elvira Palumbo, Sara Vitale, Livia Silvia Adriana Augustin, Maria Grimaldi, Emanuela Rotondo, Flavia Nocerino, Rosa Pica (Epidemiology and Biostatistics Unit, Istituto Nazionale Tumori, IRCCS-Fondazione G. Pascale, Naples, Italy); Ileana Parascandolo, Carolina De Fusco, Teresa Di Lauro, Annabella Di Martino, Martina Fontana, Ludovica Abbadessa, Anna Licia Mozzillo, Arianna Piccirillo, Vittoria Di Giacomo (Dietetics and Artificial Nutrition, Istituto Nazionale Tumori IRCCS-“Fondazione G. Pascale”, 80131 Naples, Italy); Michelino De Laurentiis, Francesco Auriemma, Ludovica Orsi, Adele Tatarella (Department of Breast and Thoracic Oncology, Istituto Nazionale Tumori, IRCCS-Fondazione G. Pascale, Naples, Italy); Francesco Perri, Vincenza Liccardo, Claudia Olivetta (Medical and Experimental Head and Neck Oncology Unit, Istituto Nazionale Tumori, IRCCS-Fondazione G. Pascale, Naples, Italy); Antonio Daniele, Pasqualina Perrotta (Experimental Clinical Abdominal Oncology Unit, Istituto Nazionale Tumori IRCCS-Fondazione G. Pascale, Naples, Italy); Alessandro Morabito, Simona Damiano, Amalia Illiano (Thoracic Medical Oncology, Istituto Nazionale Tumori IRCCS-Fondazione G. Pascale, Naples, Italy); Paolo Antonio Ascierto, Assunta Calignano (Melanoma Cancer Immunotherapy and Innovative Therapy Unit, Istituto Nazionale Tumori IRCCS-Fondazione G. Pascale, Naples, Italy); Rosa Tambaro, Sabrina Rossetti, Marilena Di Napoli, Carmela Pisano, Sabrina Chiara Cecere, Giuseppina Canciello, Gelsomina Iovane, Rossella Noce, Lucia Formisano, and Rosaria Grimaldi (Department of Urology and Gynecology, Istituto Nazionale Tumori IRCCS-Fondazione G. Pascale, Naples, Italy); Tiziana Stallone (Ente Nazionale di Previdenza e Assistenza a favore dei Biologi”—ENPAB, Rome, Italy).
