# Supplementary material for: Exploring Body Composition Profiles in Patients with Gastrointestinal Cancers: A Cross-Sectional Analysis of the NUTRISCREEN Project
Source: Curr Oncol. 2026 Sep 30;33(10):591. doi: 10.3390/curroncol33100591 (PMC13703113; doi:10.3390/curroncol33100591)

Supplementary materials

Exploring Body Composition Profiles in Patients with Gastrointestinal Cancers: a Cross-sectional Analysis of the NUTRISCREEN Project

Figure s1

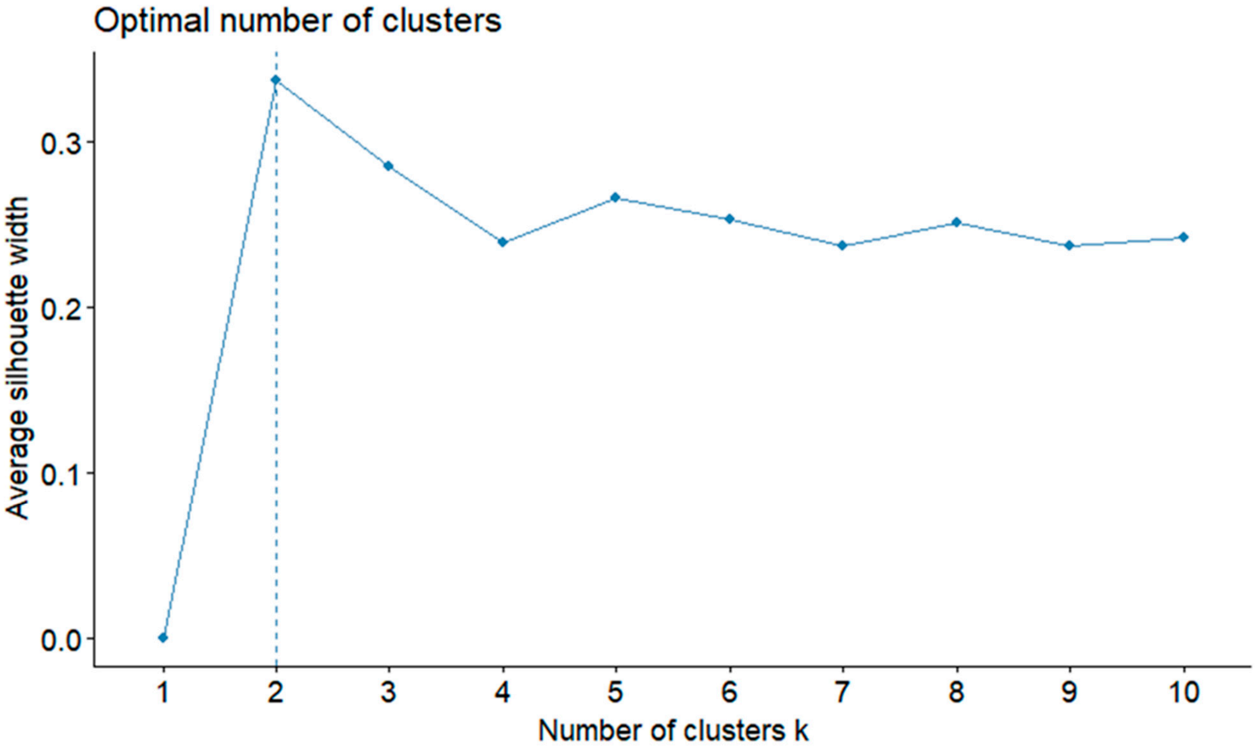

Supplement: Supplementary file 1 [file curroncol-33-00591-s001.zip › curroncol-4519494-supplementary.pdf]
